# Synaptic localization of C9orf72 regulates post-synaptic glutamate receptor 1 levels

**DOI:** 10.1186/s40478-019-0812-5

**Published:** 2019-10-24

**Authors:** Shangxi Xiao, Paul M. McKeever, Agnes Lau, Janice Robertson

**Affiliations:** 1 Tanz Centre for Research in Neurodegenerative Diseases, University of Toronto, Krembil Discovery Tower, 60 Leonard Ave., 4th floor - 4KD-481, Toronto, ON M5T 0S8 Canada; 2 Department of Laboratory Medicine and Pathobiology, University of Toronto, Toronto, ON Canada

**Keywords:** Amyotrophic lateral sclerosis, Frontotemporal dementia, C9orf72, PSD-95, GluR1, Rab39b

## Abstract

A hexanucleotide repeat expansion in a noncoding region of *C9orf72* is the most common genetic cause of amyotrophic lateral sclerosis (ALS) and frontotemporal dementia (FTD). Reduction of select or total C9orf72 transcript and protein levels is observed in postmortem C9-ALS/FTD tissue, and loss of *C9orf72* orthologues in zebrafish and *C. elegans* results in motor deficits. However, how the reduction in C9orf72 in ALS and FTD might contribute to the disease process remains poorly understood. It has been shown that C9orf72 interacts and forms a complex with SMCR8 and WDR41, acting as a guanine exchange factor for Rab GTPases. Given the known synaptosomal compartmentalization of C9orf72-interacting Rab GTPases, we hypothesized that C9orf72 localization to synaptosomes would be required for the regulation of Rab GTPases and receptor trafficking. This study combined synaptosomal and post-synaptic density preparations together with a knockout-confirmed monoclonal antibody for C9orf72 to assess the localization and role of C9orf72 in the synaptosomes of mouse forebrains. Here, we found C9orf72 to be localized to both the pre- and post-synaptic compartment, as confirmed by both post-synaptic immunoprecipitation and immunofluorescence labelling. In C9orf72 knockout (C9-KO) mice, we demonstrated that pre-synaptic Rab3a, Rab5, and Rab11 protein levels remained stable compared with wild-type littermates (C9-WT). Strikingly, post-synaptic preparations from C9-KO mouse forebrains demonstrated a complete loss of Smcr8 protein levels, together with a significant downregulation of Rab39b and a concomitant upregulation of GluR1 compared with C9-WT mice. We confirmed the localization of Rab39b downregulation and GluR1 upregulation to the dorsal hippocampus of C9-KO mice by immunofluorescence. These results indicate that C9orf72 is essential for the regulation of post-synaptic receptor levels, and implicates loss of C9orf72 in contributing to synaptic dysfunction and related excitotoxicity in ALS and FTD.

## Introduction

Amyotrophic lateral sclerosis (ALS) is a fatal adult-onset neurodegenerative disease primarily affecting motor neurons of the motor cortex, brain stem, and spinal cord. With no effective treatment, disease course is typically rapid, resulting in complete paralysis and death within 2–5 years after diagnosis. Hexanucleotide (G4C2) repeat expansions within the first intron of *C9orf72* are the most common known genetic cause of both ALS and frontotemporal dementia (FTD) [14, 40]. Initial reports on *C9orf72* expansions indicated that a length of > 30 was pathogenic; however, there have been several cases where 30–70 repeats do not result in disease, indicating there is no discernible pathological cut-off [17, 35, 59, 60]. As a result, how the expanded G4C2 repeats in *C9orf72* cause neurodegeneration in ALS and FTD remains largely uncertain. Three potential pathomechanisms have been proposed to result from the repeat expansions [21, 30, 52]: (1) RNA-mediated toxicity through sequestration of RNA-binding proteins in nuclear repeat RNA foci; (2) accumulation of five dipeptide repeat (DPR) proteins, glycine-alanine (GA), glycine-arginine (GR), proline-alanine (PA), proline-arginine (PR), and glycine-proline (GP), by repeat-associated non-ATG (RAN) translation; and (3) loss of function through *C9orf72* haploinsufficiency.

Evidence from human tissues, and cell and animal models has demonstrated that RNA foci are generated in neural cells and the G4C2 repeat structures sequester RNA-binding proteins [1, 14, 15, 17, 37, 50, 63]. In addition, it has been shown that GA, GR, PA, PR, and GP differentially accumulate across different brain regions in ALS/FTD patients [2, 3, 18, 38, 39, 42, 54]. However, evidence has indicated that the distribution of RNA foci and DPRs only show a minor relationship with the severity of neurodegeneration across brain regions, and DPR inclusions in disease are rarely observed in motor neurons at autopsy [12, 13, 32, 33]. Indeed, a recent discovery demonstrated that somatic expansion of the G4C2 repeats does not occur in ALS spinal cord tissues [41]. Interestingly, one group reported an ALS patient presenting with behavioural variant FTD who carried a loss-of-function splice site mutation (c.601 -2A > G) that created a premature stop codon (p.I201fsX235), resulting in reduced C9orf72 mRNA levels in leukocytes relative to control cases [31]. We recently reported a 90-year-old individual carrying 70 G4C2 repeats who was neurologically asymptomatic at autopsy and who had widespread accumulation of RNA foci and DPRs in the brain, but had increased C9orf72 protein levels and no TDP-43 pathology [35, 59]. These findings emphasize the importance of assessing the contribution of C9orf72 protein levels to disease mechanism. To date, reduced expression of select or total C9orf72 transcripts [1, 6, 14, 20] or its protein level [57, 61] in C9orf72 G4C2 repeat carrier-derived cells or postmortem tissues from C9-ALS/FTD patients have been widely reported. In animal models, knockdown or deletion of C9orf72 orthologues cause motor phenotypes in zebrafish [9] and *C. elegans* [53], respectively. However, loss of C9orf72 in mice does not induce motor neuron deficits, nor does it produce TDP-43 proteinopathy [24, 27]. Collectively, a full understanding of C9orf72 function is needed to elucidate its contribution to the disease mechanism.

Sequence and structure analyses have shown that C9orf72 shares homology with DENN (differentially expressed in normal and neoplastic cells) domain proteins [23, 29, 66], which are Rab GTPase guanine exchange factors (GEFs) [34, 65]. C9orf72 forms a complex with Smcr8 and Wdr41 and can act as a GEF for Rab8 and Rab39b [11, 44, 62, 64]. Given that many Rabs show a synaptosomal distribution [26] and that two studies have shown C9orf72 enrichment at synapses [4, 16], we hypothesized that the loss of C9orf72 would lead to alterations in Rab family interactors and glutamatergic receptor levels in synaptosomes. To further understand the biochemical distribution of C9orf72 in the brain, we combined synaptosomal [51] and post-synaptic density preparations [8, 10] with a knockout-confirmed monoclonal antibody for C9orf72, which shows both biochemical and immunohistochemical specificity for C9orf72 [28], to assess the distribution of C9orf72 at synaptosomes. Here, we have demonstrated that the mouse ortholog for C9orf72 (31100432021Rik) is localized to both the pre- and post-synapses and is essential for stable Rab39b and GluR1 levels in post-synaptic densities.

## Materials and methods

### Mouse breeding

All animal protocols were conducted in accordance with the Canadian Council on Animal Care and approved by the University of Toronto Animal Care Committee. Knockout C9orf72 mice were obtained through a generous gift from Dr. Don Cleveland (UCSD) and Dr. Clothilde Lagier-Tourenne (UMass). Breeding and genotyping were conducted as previously described [24]. Briefly, C9orf72 heterozygous mice (C57BL/6 background) were crossed, resulting in the production of homozygous C9orf72 KO (C9-KO) and wild-type (C9-WT) littermates. For all biochemistry and immunohistochemical experiments, 3-month old mice were empirically selected.

### Post-synaptic density fractionation

**Fig. 1:**
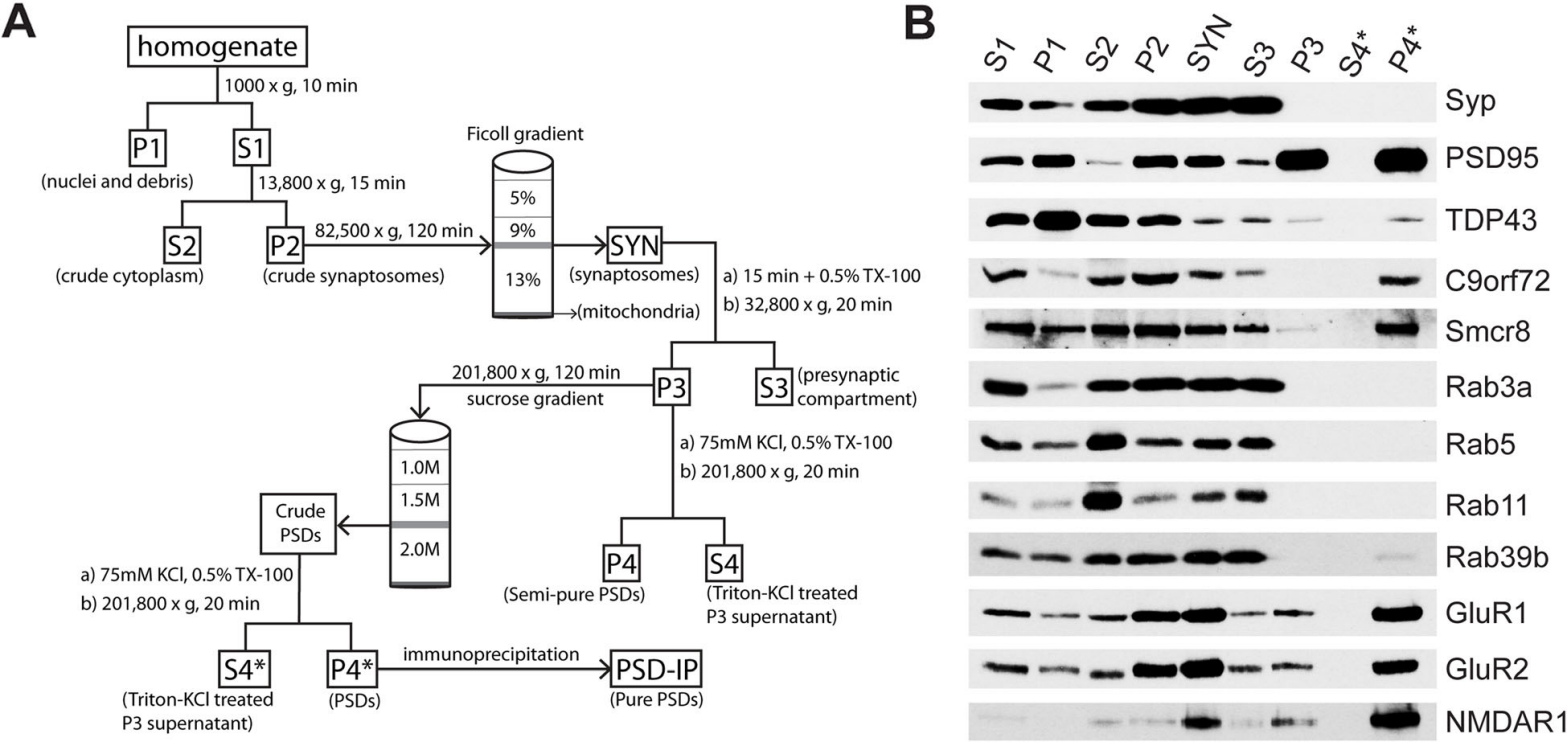
Biochemical distribution of C9orf72 and Rab proteins in the pre- and post-synaptic compartments of wild-type murine forebrains. **a** Flowchart for the fractionation of synaptosomes, pre-synapses, and post-synaptic densities (PSDs) used in the current study. S1 = post-nuclear supernatant; P1 = nucleus and debris; S2 = crude cytoplasm; P2 = crude synaptosomes; SYN = pure synaptosomes; S3 = presynaptic terminals; P3 = crude PSDs; S4 = Triton X-100-KCl treated P3 supernatant; P4 = semi-pure PSDs; S4* = Triton X-100-KCl treated crude PSD supernatant; P4* = PSDs; PSD-IP = pure PSDs. **b** Distribution of markers across synaptic fractions in C57BL/6 mice. Synaptophysin (Syp); Post-Synaptic Density Protein 95 (PSD-95); TAR DNA Binding Protein 43 (TDP-43); Chromosome 9 open reading frame 72 (C9orf72); Smith-Magenis syndrome Chromosomal Region candidate gene 8 (Smcr8); Ras-related proteins Rab3a, Rab5, Rab11, Rab39b; Glutamate receptor 1 (GluR1); glutamate receptor 2 (GluR2); N-methyl-D-aspartic acid receptor 1 (NMDAR1)

The fractionation of intact synaptosomes, followed by postsynaptic densities (PSDs), was performed as previously described [8, 51] with some adaptations (Fig. 1a). For the characterization of the distribution of protein markers in the PSD fraction of C57BL/6 WT mice, we used three biological replicates (*n* = 12 mice per replicate). Mice were euthanized with controlled flow CO_2_ followed by cervical dislocation. The brains were rapidly removed from the skull and then the cerebellum and brainstem from each brain were discarded, leaving dissected forebrains for each mouse. Mouse forebrains were homogenized with a Dounce homogenizer in ice-cold homogenization buffer (HB; 320 mM sucrose, 5 mM HEPES [pH 7.4]) and then centrifuged at 1000×*g* for 10 min at 4 °C. The resulting pellet was homogenized with RIPA buffer (150 mM NaCl, 50 mM Tris-HCl [pH 7.4], 1% w/v TX-100, 0.5% w/v sodium deoxycholate, 1 mM EDTA, 0.1% SDS) and saved as the post-nuclear/debris fraction (P1). The supernatant (S1) was centrifuged at 13,800×*g* for 15 min at 4 °C. The supernatant (S2) was saved as the cytoplasmic fraction and the pellet (P2) was resuspended in ice-cold HB and then loaded onto a discontinuous Ficoll gradient (13, 9, 5% w/v) prepared in HB and centrifuged at 82,500×*g* for 120 min at 4 °C. Intact synaptosomes (SYN) were retrieved at the boundary between 9 and 13% in the Ficoll gradient and rinsed two times with HB. The resulting pellet was resuspended in cold resuspending buffer (RB; 0.32 M sucrose, 1 mM NaHCO_3_) and then lysed in an equal volume of lysis buffer 1 (LB1; 1% Triton X-100, 12 mM Tris-HCl [pH 8.1]). The lysed synaptosomes were end-over-end rotated for 15 min at 4 °C and centrifuged at 32,800×*g* for 20 min. The supernatant was saved as the pre-synaptic compartment (S3), whereas the pellet (P3) was resuspended in RB, loaded onto a sucrose gradient (2.0 M, 1.5 M, 1.0 M), and then centrifuged at 201,800×*g* for 120 min. The crude PSDs were retrieved from the 1.5–2.0 M sucrose interface and resuspended in RB, followed by treatment with equal parts lysis buffer 2 (LB2; 150 mM KCl, 1% TX-100). After end-over-end rotation for 15 min at 4 °C, the crude PSD solution was centrifuged at 201,800×*g* for 4 °C. The supernatant was saved as the Triton-KCl treated fraction (S4*), whereas the pellet was resuspended in RIPA buffer and sonicated at < 5 watts to isolate PSDs (P4*).

To allow for the direct comparison of C9-WT versus C9-KO mice (*n* = 2 per sample; *n* = 4 biological replicates), the sucrose gradient step was omitted after resuspension of the P3 fraction, as shown in Fig. 1a. We implemented this approach since it was empirically determined that all markers identified in PSDs were present in semi-pure PSDs. For the C9-WT versus C9-KO experiments, P3 was treated directly with RB along with addition of LB2, as described above, followed by end-over-end rotation for 15 min at 4 °C. Centrifugation was then performed at 201,800×*g* for 4 °C, where the supernatant was saved as the LB2-treated fraction (S4) and the pellet was resuspended in RIPA buffer and then sonicated (< 5 W) to obtain semi-pure PSDs (P4). Protein concentrations for each fraction were estimated using the bicinchoninic acid assay for electrophoretic loading.

### PSD-95 immunoprecipitation

For the immunoprecipitation (IP) experiment, the procedure for isolating PSDs was performed as described above for the characterization of protein marker distribution in PSDs (Fig. 1a). We adapted our IP strategy based on an affinity-based approach in a previous study [56]. Briefly, Protein A magnetic beads (Surebeads 161–4023, BioRad) were washed two times with phosphate buffered saline (PBS, 137 mM NaCl, 2.7 mM KCl, 4.3 mM Na_2_HPO_4_, 1.47 mM KH_2_PO_4_, pH 7.4). Next, washed beads were incubated with either polyclonal PSD-95 antibody (ab18258) or rabbit IgG (#2729, Cell Signaling Technology) at 1:200 dilution in PBS for 1 h at ambient temperature. Beads with α-PSD-95 or rabbit IgG bound were then washed with Tris-buffered saline (TBS; 50 mM Trizma base, 150 mM NaCl, pH 7.6) with 0.1% Tween 20 three times for 5 min. Pre-sonicated P4 fractions were incubated with either PSD-95 antibody-coated beads or rabbit-IgG-coated beads for 2 h at 4 °C. Next, beads were collected by magnetic rack and washed three times for 3 min in TBS with 0.1% Tween 20. Elution was performed in RIPA buffer with sample buffer added (3X; 187.5 mM Trizma base, 6% SDS, 30% glycerol, 0.03% bromophenol blue, 15% β-mercaptoethanol, pH 7.6) with a 5 min incubation at 95 °C.

### Gel electrophoresis and immunoblotting

Samples were solubilized in sample buffer (3X) and boiled at 95 °C for 5 min for immunoblotting. All samples were electrophoresed on 10% (w/v) sodium dodecyl sulphate-polyacrylamide (SDS-PAGE) gels. Separated proteins were transferred to polyvinylidene fluoride (PVDF) membranes. For all immunoblot experiments, membranes were blocked for 1 h at ambient temperature in blocking buffer composed of 5% (w/v) skim milk powder in TBS. PVDF membranes were incubated overnight at 4 °C with primary antibodies. A complete list of primary antibodies and the appropriate methodology used is shown in Additional file 1: Table S1. Membranes were then washed with TBS containing 0.05% Tween 20 and incubated for 1 h at ambient temperature with the following secondary antibodies diluted in blocking buffer: either α-mouse horseradish peroxidase (HRP)-conjugated (NA931, GE Healthcare; 1:5000), α-rabbit HRP-conjugated (NA934, GE Healthcare, 1:5000), or α-goat HRP-conjugated (sc-2433, Santa Cruz, 1:5000) antibody. Visualization of immunoblot labelling was achieved using chemiluminescence with the Western Lighting Plus ECL kit (Perkin Elmer).

### Immunoblot image analysis and quantification

Densitometric analysis of immunoblots was performed using the ImageJ distribution Fiji [43]. Protein levels were estimated for each lane by calculating the relative density using an appropriate marker for that fraction (Synaptohysin [Syp] for SYN and S3; PSD-95 for P4). Bar plots were used for visualization of relative protein levels and the statistical analysis comparing C9-WT and C9-KO mice was performed with Prism (v8.0.2, GraphPad). For this, multiple t-tests were performed, and the false discovery rate was controlled using the Benjamini, Krieger, and Yekutieli procedure (Q = 1%).

### Immunofluorescence

Mice were anaesthetized with ketamine/xylazine (1 mg/g) by intraperitoneal injection. For C9orf72/PSD-95 staining, transcardial perfusion was performed with phosphate buffered saline (PBS; 137 mM NaCl, 2.7 mM KCl, 4.3 mM Na_2_HPO_4_, 1.47 mM KH_2_PO_4_, pH 7.4) followed by 10% neutral buffered formalin (HT501128, Sigma-Aldrich). Brains were removed, post-fixed in formalin for exactly 24 h at ambient temperature, then placed in 70% ethanol for 1 week prior to processing and embedding in paraffin blocks. Formalin-fixed, paraffin-embedded mouse tissue blocks from C9-KO (*n* = 3) and C9-WT (*n* = 3) mice were sectioned sagittally at 6 μm and mounted on positively-charged slides. Deparaffinization was performed by placing sections on a 60 °C heat block for 20 min, in xylene (3 × 5 min), 50:50 xylene:ethanol, and then through graded ethanol washes (100, 95, 75, 50% w/v) prior to pure water. Heat-induced antigen retrieval was achieved using Tris-EDTA buffer (10 mM Trizma base, 1 mM EDTA, 0.1% Tween 20, pH 9.0) at 110 °C for 15 min in a pressure cooker. Blocking was performed with 10% donkey serum (EMD Millipore) and 0.3% TX-100 in TBS for 1 h at ambient temperature. Primary antibody incubation was performed overnight at 4 °C for C9orf72 (GTX634482; Genetex) and either PSD-95 (61–5900, Invitrogen), Synaptoporin (102,002, Synaptic Systems), or Rab39b (12162–1-AP, Proteintech) diluted in DAKO Antibody Diluent (S0809; Agilent). After 3 × 20 min washes in TBS + 0.1% Tween 20 (TBST), secondary incubation was performed at ambient temperature with donkey α-rabbit 488 and donkey α-mouse 594 Alexa Fluor secondary antibodies (Invitrogen; 1:500) diluted in DAKO Antibody Diluent. Slides were washed 3 × 20 min in TBST prior to mounting with ProLong Gold antifade reagent with 4′,6-diamidino-2-phenylindole [DAPI] (P36931; Life Technologies).

For GluR1 and Rab39b labelling, transcardial perfusion was performed with ice-cold PBS followed by ice-cold 4% paraformaldehyde (PFA) in PBS. Brains were removed and post-fixed in PFA for 24 h at 4 °C. PFA-fixed brains were cryo-protected by immersion in PBS with 30% sucrose. 40 μm sagittal sections were cut on a freezing microtome (HM430, Thermo Scientific), placed in anti-freeze solution (30% glycerol, 30% ethoxyethanol, 40% PBS), and stored at − 20 °C. Sections containing the dorsal hippocampus were rehydrated in PBS and then permeablized in PBS containing 0.4% TX-100 for 20 min. Blocking was performed in 10% donkey serum (EMD Millipore) with 3% BSA in PBS containing 0.4% TX-100 for 2 h. Primary antibody incubation was performed with polyclonal rabbit α-GluR1 (AB1504; 1:200) or α-Rab39b (Proteintech; 1:2000) at 4 °C for 48 h. Following three 20 min washes with PBS containing 0.1% TX-100, sections were incubated with donkey α-rabbit 488 Alexa Fluor secondary antibody (Invitrogen; 1:500) diluted in the same blocking buffer for 2 h at ambient temperature. After three 20 min washes with PBS, sections were mounted on positively-charged slides and allowed to completely dry at ambient temperature. Mounting was then performed with ProLong Gold antifade reagent with DAPI (P36931; Life Technologies). Micrographs were captured using a Leica DMI6000B microscope with the Volocity Acquisition Suite (v6.3, Perkin Elmer).

### Micrograph image analysis and quantification

For all images, deconvolution of low and high magnification micrographs was performed with 25 iterations and a confidence level of 95% per channel using the Volocity Analysis Suite (v6.3, Perkin Elmer). For GluR1 and Rab39b, fluorescence intensity in the hippocampus of C9-WT versus C9-KO mice was calculated from exported images (*n* = 3 slices per biological replicate) using Fiji [43]. Qualitative assessment of GluR1 levels indicated increased immunofluorescence across all hippocampal subfields, so the entire hippocampal area was outlined in the fluorescence intensity measurement. For Rab39b, the intensity was measured by outlining the mossy fiber area given the empirically determined distribution in that region alone. Mean fluorescence intensity in each case was calculated by subtracting background intensity from raw intensity and then dividing by the selected hippocampal area. Statistical analyses were performed with Prism (v8.0.2, GraphPad), where the mean fluorescent intensity of GluR1 and Rab39b in C9-WT versus C9-KO were contrasted using a paired t-test (*p* < 0.05).

## Results

### C9orf72 localizes to both the pre- and post-synapses in murine forebrains

Given that different Rab proteins show unique localization to the pre- and post-synaptic compartment [22, 26], we reasoned that C9orf72 and its interacting partner Smcr8 [11, 62, 64] would be present in post-synaptic densities. To assess this, we performed biochemical fractionation of the pre- and post-synapses in mouse forebrains as described previously [8, 51] with some modifications (Fig. 1a). With this approach, we showed enrichment for the pre-synaptic vesicular marker synaptophysin (Syp) in synaptosomes (SYN) and the pre-synaptic compartment (S3), whereas Syp was absent in the post-synaptic fraction (P4*) (Fig. 1b). The established post-synaptic density marker PSD-95 was enriched in the expected fractions (SYN, P3, and P4*) and depleted in the pre-synapses (S3) (Fig. 1b). In addition, post-synaptic receptors GluR1, GluR2, and NMDAR1 were also enriched in P4* (Fig. 1b). We also detected pre- and post-synaptic TDP-43 in S3 and P4* (Fig. 1b) and found that Rab3a, Rab5, and Rab11 were enriched in the S3 fraction, but depleted in P4* (Fig. 1b). However, Rab39b was found in both the pre- and post-synaptic compartments (Fig. 1b), confirming the differential synaptic distribution of Rab family proteins in the brain [22, 26]. Using a knockout-confirmed mouse monoclonal antibody against C9orf72 [28], we detected an ~ 52 kDa band for C9orf72 corresponding to the long isoform of the protein [61] in S3 and P4* (Fig. 1b). We additionally found the C9orf72 interacting partner Smcr8 in S3 and P4* (Fig. 1b), indicating both a pre- and post-synaptic localization of C9orf72 and Smcr8.

**Fig. 2:**
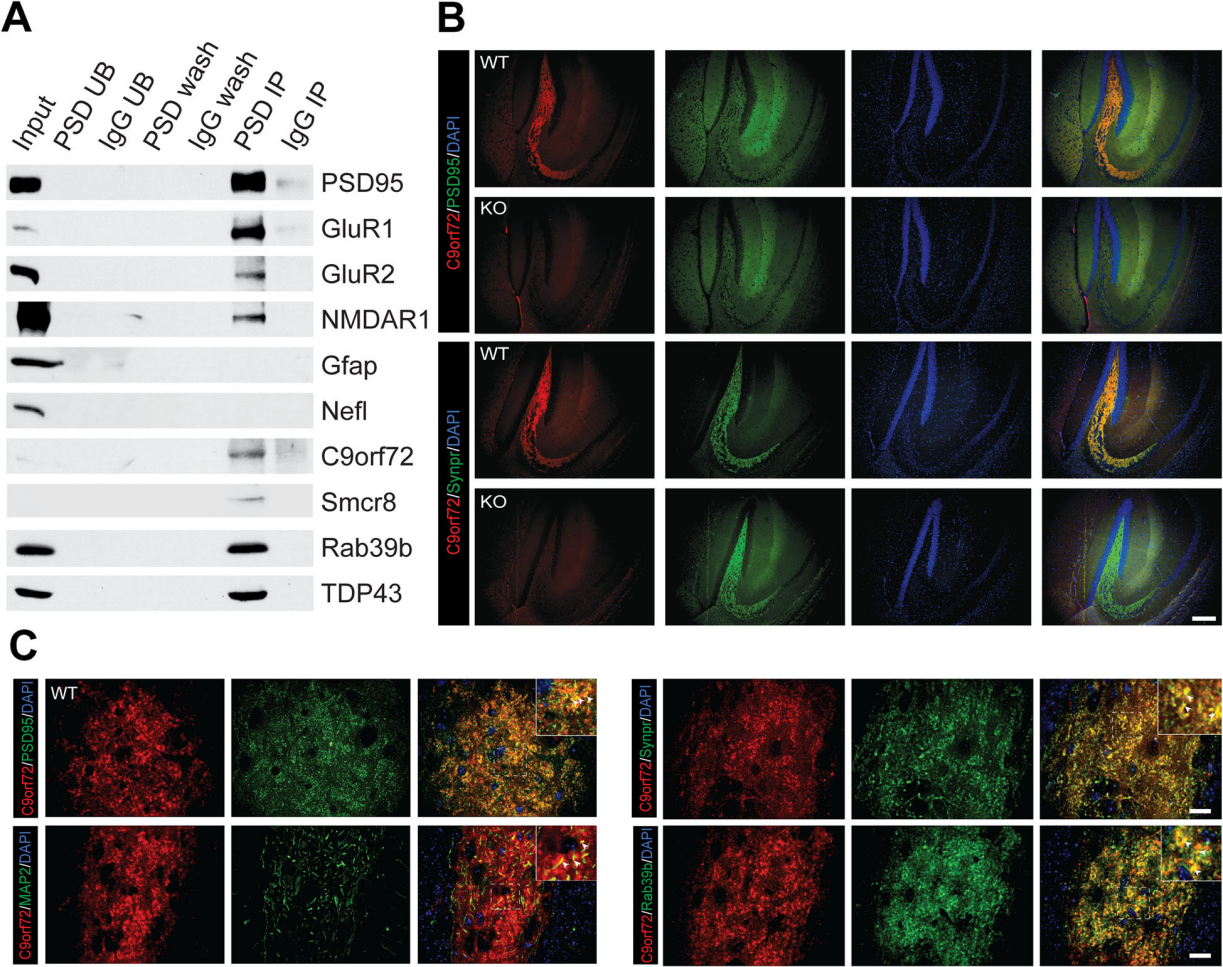
C9orf72 and Rab39b are localized to post-synaptic densities. **a** Immunoblot analysis of affinity purified PSD-95 complexes from C9-WT mice. PSD = PSD-95 antibody; UB = unbound fraction; IgG = rabbit IgG; wash = last wash. Synaptophysin (Syp); Post-Synaptic Density Protein 95 (PSD95); TAR DNA Binding Protein 43 (TDP-43); Chromosome 9 open reading frame 72 (C9orf72); Smith-Magenis syndrome Chromosomal Region candidate gene 8 (Smcr8); Ras-related proteins Rab3a, Rab5, Rab11, Rab39b; Glutamate receptor 1 (GluR1); glutamate receptor 2 (GluR2); N-methyl-D-aspartic acid receptor 1 (NMDAR1). Glial fibrillary acid protein (Gfap); Neurofilament Light Chain (Nefl). **b** Low magnification immunofluorescent micrographs of C9orf72, PSD-95, and DAPI or C9orf72, Synaptoporin (Synpr), and DAPI in the dorsal hippocampus of C9-WT and C9-KO mice. Scale = 200 μm. **c** High magnification immunofluorescent micrographs of C9orf72 with either PSD-95, microtubule associated protein 2 (MAP2), Synpr, or Rab39b in the CA3 region of the hippocampus from C9-WT and C9-KO mice. Insets contain arrowheads which indicate co-detection of fluorescent signal between the red and green channels. Scale = 20 μm

To validate C9orf72 presence in the post-synaptic density, we performed immunoprecipitation (IP) of the PSD-95 complex on 12 pooled wild-type mouse forebrains, as previously described [56] with some alterations (Fig. 1a). Both the input (P4*) and IP fractions enriched for the bait protein PSD-95 (Fig. 2a). Post-synaptic receptors GluR1, GluR2, and NMDAR1, in addition to TDP-43, immunoprecipitated with PSD-95 (Fig. 2a), whereas the established contaminants neurofilament light chain (Nefl) and glial fibrillary acidic protein (Gfap) [56] were absent from the PSD IP (Fig. 2a). Notably, C9orf72 was detected in the PSD IP, along with its interacting partners Smcr8 and Rab39b (Fig. 2a). To confirm the brain region localization of synaptic C9orf72, we performed double immunofluorescence labelling with antibody to C9orf72 with either a pre- (Synpr) or post-synaptic (PSD-95) marker on sagittal sections in C9-WT and C9-KO mice. Low magnification images indicated that C9orf72 was present in the forebrain regions that we and others previously identified [16, 28], including mossy fiber synapses of the hippocampus (Fig. 2b), the glomerular layer of the olfactory bulb, synapses of the basal ganglia, substantia nigra, and inferior olive, and in the granular layer of the cerebellum (data not shown). We detected partial overlap of C9orf72 with both pre-synaptic (Synpr) and post-synaptic (PSD-95) markers in the mossy fiber area of the dorsal hippocampus, whereas this synaptic C9orf72 signal was ablated in C9-KO mice (Fig. 2b). Deconvoluted, high magnification images from C9-WT mossy fiber synapses demonstrated partial overlap of C9orf72 with pre-synaptic (Synpr), post-synaptic (PSD-95), and dendritic (MAP 2) proteins (Fig. 2c). Furthermore, high-powered micrographs also confirmed that C9orf72 was partially co-localized with Rab39b in the mossy fiber synapse region of C9-WT mice (Fig. 2c), indicating that C9orf72 shares synaptic localization with previously identified interactors.

### Synaptosomal and pre-synaptic preparations are unchanged in C9-KO versus C9-WT mice

**Fig. 3:**
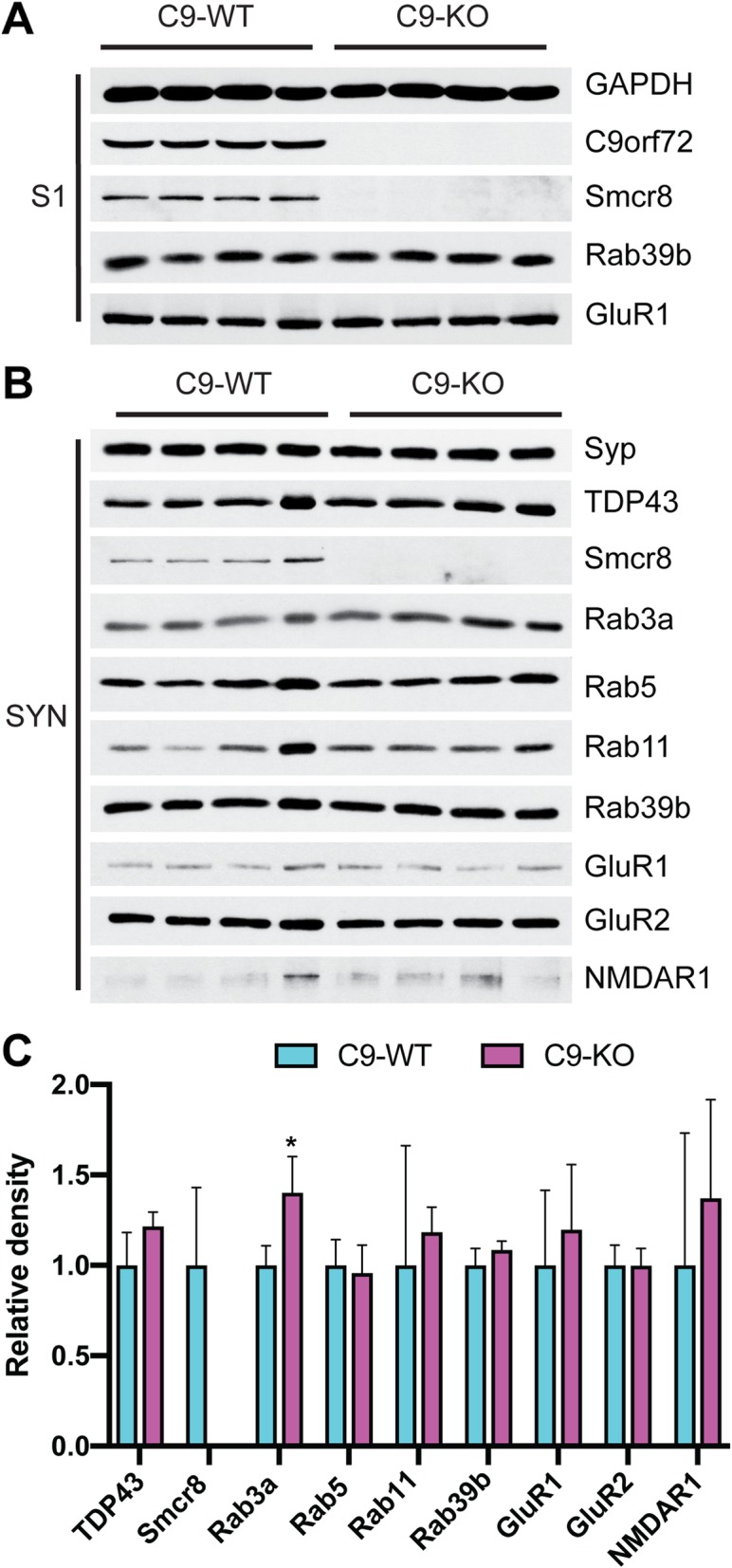
Rab3a protein levels are increased in the synaptosomal fraction of C9-KO mice relative to C9-WT mice. **a** Immunoblots from the post-nuclear input fraction (S1) from C9-WT and C9-KO mice (*n* = 2 mice per sample; *n* = 4 biological replicates per group). Loading confirmed by glyceraldehyde 3-phosphate dehydrogenase (GAPDH); Chromosome 9 open reading frame 72 (C9orf72); Smith-Magenis syndrome Chromosomal Region candidate gene 8 (Smcr8); Ras-related protein Rab39b; Glutamate receptor 1 (GluR1). **b** Immunoblots from the synaptosomal (SYN) fraction. **c** Bar plots of mean immunoblot band density for each antibody probed in the SYN fraction. x-axis = C9-WT versus C9-KO for each antibody; y-axis = relative density (ratio relative to wild-type); error bars = standard deviation; *Q<0.01. Synaptophysin (Syp); Post-Synaptic Density Protein 95 (PSD95); TAR DNA Binding Protein 43 (TDP-43); Rab family members Rab3a, Rab5, Rab11, Rab39b; Glutamate receptor 1 (GluR1); glutamate receptor 2 (GluR2); N-methyl-D-aspartic acid receptor 1 (NMDAR1)

**Fig. 4:**
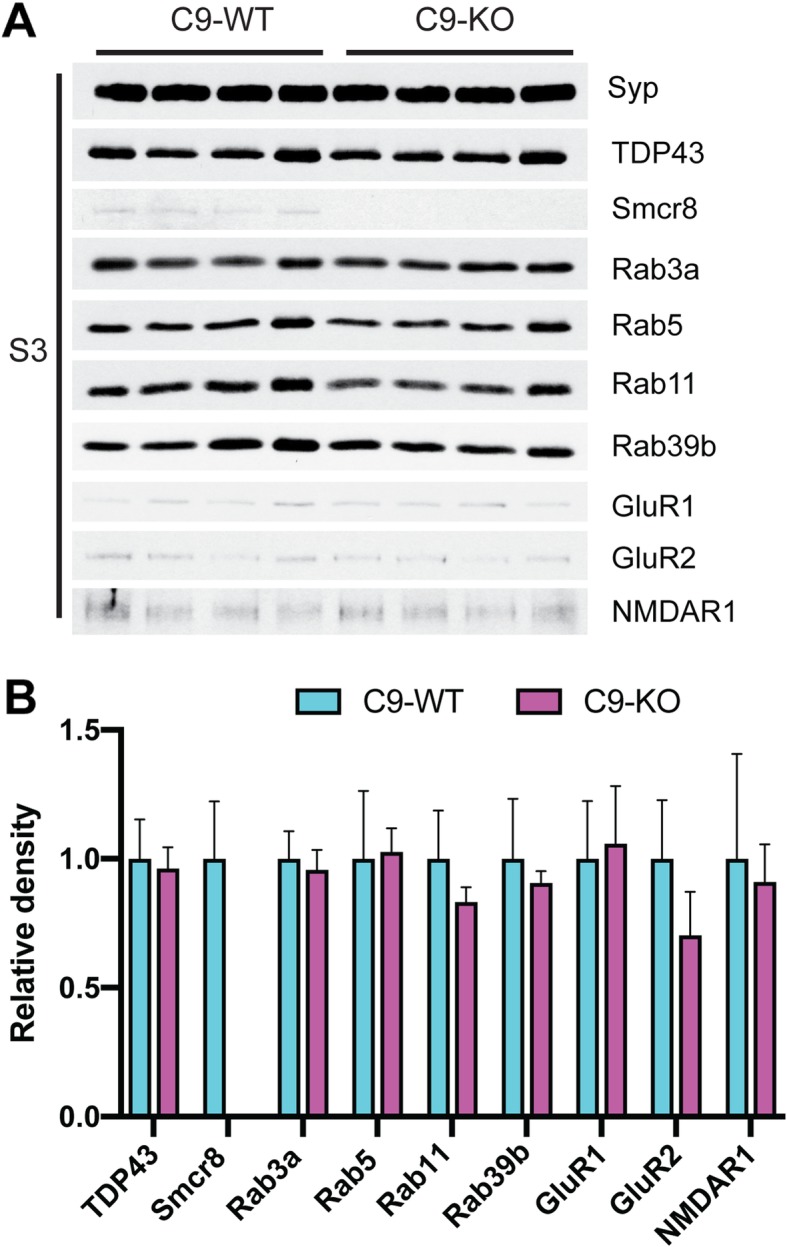
Pre-synaptic fraction shows stable protein levels between C9-WT and C9-KO mice. **a** Immunoblots from the pre-synaptic compartment (S3) from C9-WT and C9-KO mice (*n* = 2 mice per sample; *n* = 4 biological replicates per group). **b** Bar plots of mean S3 immunoblot band densities for each antibody probed in the S3 fraction. y-axis = relative density; error bars = standard deviation. Synaptophysin (Syp); TAR DNA Binding Protein 43 (TDP-43); Chromosome 9 open reading frame 72 (C9orf72); Smith-Magenis syndrome Chromosomal Region candidate gene 8 (Smcr8); Ras-related proteins Rab3a, Rab5, Rab11, Rab39b; Glutamate receptor 1 (GluR1); glutamate receptor 2 (GluR2); N-methyl-D-aspartic acid receptor 1 (NMDAR1)

Given that C9orf72 localized to both pre- and post-synaptic regions, we hypothesized that Rab family members, Smcr8, and synaptic receptor protein levels would show alterations in C9-KO versus C9-WT mice. We purified intact synaptosome from C9-WT and C9-KO mouse forebrains and then released the pre-synaptic compartment using Triton X-100 (S3) (Fig. 1a). Consistent protein loading was shown by stable GAPDH levels across input fractions (S1) in C9-WT and C9-KO mice. As expected, C9orf72 was absent in S1 from C9-KO mice along with undetectable Smcr8 protein levels (Fig. 3a), which is in line with previous C9orf72 knockout studies in vitro [67] and in vivo [55]. However, Rab39b and GluR1 were unchanged between C9-WT and C9-KO in the S1 fraction (Fig. 3a), which was verified by densitometric quantification (Additional file 1: Figure S1). In synaptosomal (SYN) fractions, we detected stable levels of TDP-43, Rab5, Rab11, Rab39b, GluR1, GluR2, and NMDAR1 between C9-WT and C9-KO mice (Fig. 3b), which was quantified by densitometric analysis from Syp-corrected SYN preparations (Fig. 3b, c). Interestingly, Rab3a levels were significantly increased in C9-KO synaptosomes compared with C9-WT (Q < 0.01; Fig. 3b, c), indicating that loss of C9orf72 expression in C9-KO mice results in a compensatory increase in Rab3a levels in the pre-synaptic compartment. However, we found that Rab3a levels were unchanged in S3 between C9-WT and C9-KO mice (Fig. 4a), which we further confirmed by densitometric quantification (Fig. 4b). In the S3 fraction, the levels of TDP-43, Rab5, Rab11, Rab39b, GluR1, GluR2, and NMDAR1 were also unchanged between C9-WT and C9-KO mice (Fig. 4a, b), indicating that pre-synaptic Rab family members show stable levels in the absence of C9orf72.

### Loss of C9orf72 results in decreased Rab39b and increased GluR1 in the hippocampus of C9-KO mice

**Fig. 5:**
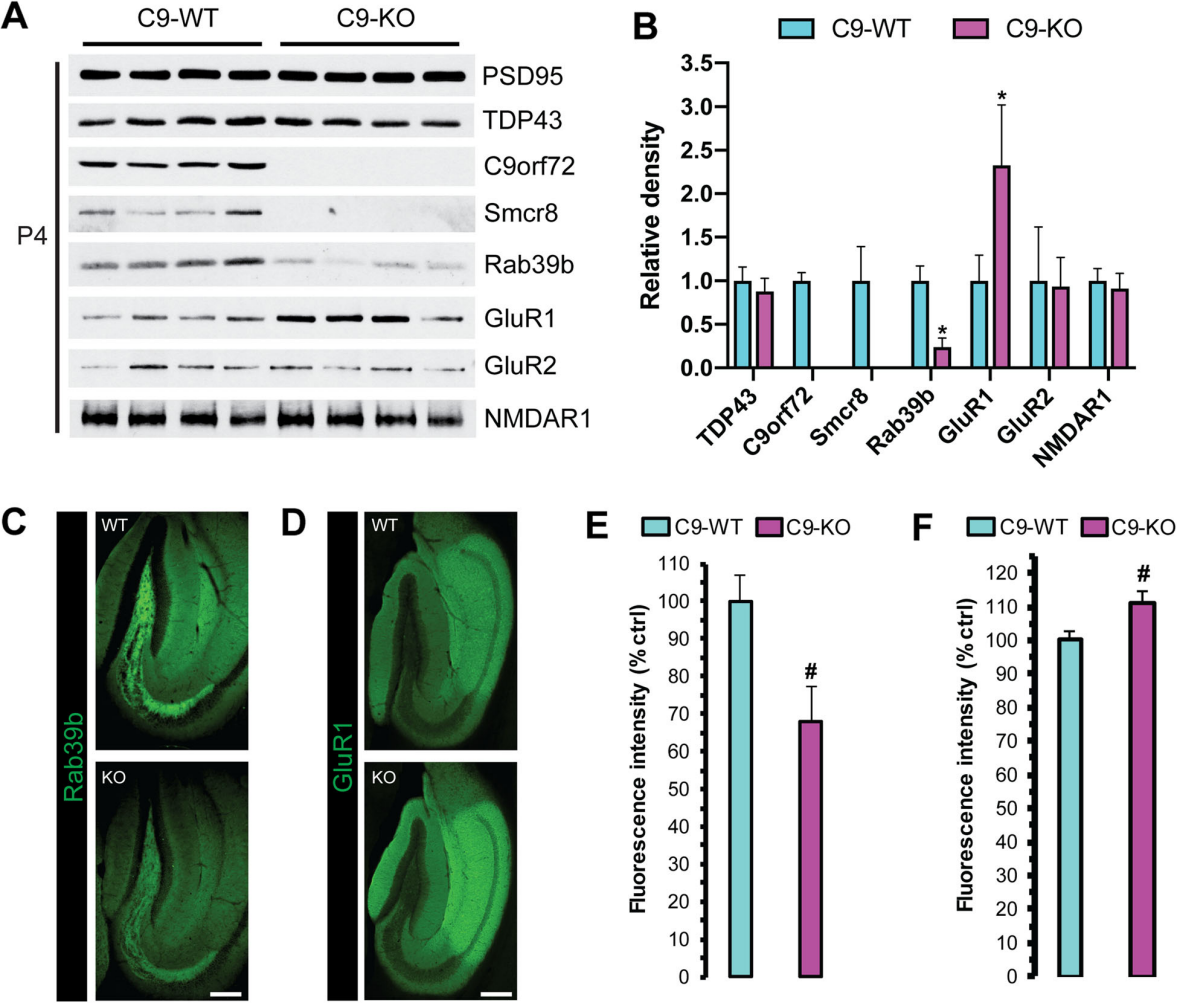
Rab39b and GluR1 levels are reciprocally altered in the post-synaptic compartment of C9-KO mice. **a** Western blot analysis of pre-synaptic density fractions (P4) from C9-WT and C9-KO mice (*n* = 2 mice per sample; *n* = 4 biological replicates per group). Post-Synaptic Density Protein 95 (PSD95); TAR DNA Binding Protein 43 (TDP-43); Chromosome 9 open reading frame 72 (C9orf72); Smith-Magenis syndrome Chromosomal Region candidate gene 8 (Smcr8); Ras-related proteins Rab39b; Glutamate receptor 1 (GluR1); glutamate receptor 2 (GluR2); N-methyl-D-aspartic acid receptor 1 (NMDAR1). **b** Bar plots of mean immunoblot band density for each antibody probed in the P4 fraction. x-axis = C9-WT versus C9-KO for each antibody; y-axis = relative density (ratio relative to wild-type); error bars = standard deviation; *Q<0.01. Representative immunofluorescent micrographs of **c** Rab39b and **d** GluR1 in the dorsal hippocampus of C9-WT versus C9-WT mice. Scale = 200 μm. Quantification of **e** Rab39b and **f** GluR1 fluorescence intensity in the dorsal hippocampus of C9-WT versus C9-WT mice (*n* = 4 animals; *n* = 3 micrographs per animal); y-axis = relative fluorescence intensity as a percentage (%) of C9-WT #p<0.001 for Rab39b; #p<0.05 for GluR1

We asked whether loss of C9orf72 would lead to alterations in post-synaptic Rab39b and receptor protein levels. For this we performed an immunoblot analysis on semi-pure post-synaptic density preparations (P4; Fig. 1a) from C9-WT and C9-KO mice. We detected stable PSD-95 between C9-WT and C9-KO mice (Fig. 5a), which we verified by densitometry, and then used PSD-95 protein levels to estimate relative abundance for the other markers screened in P4 (Fig. 5b). We observed stable TDP-43, GluR2, and NMDAR1 levels in P4 and undetectable Smcr8 levels in C9-KO preparations (Fig. 5a, b), whereas the relative amount of Rab39b was decreased and GluR1 was increased in P4 from C9-KO versus C9-WT mice (Q < 0.01; Fig. 5a, b). We next assessed the immunofluorescence intensity of Rab39b and GluR1 in 40 μm free-floating sections from C9-WT and C9-KO mouse brains. There was a concomitant decrease in Rab39b (Fig. 5c) with upregulated GluR1 protein levels (Fig. 5d) in the hippocampus of C9-KO versus C9-WT mice. After quantification, we found a significant decrease in Rab39b fluorescence intensity in the mossy fiber synapse region of C9-KO over C9-WT mice (*p* < 0.001; Fig. 5e), in agreement with the changes found by immunoblot in P4 (Fig. 5a). We did not detect a significant decrease of Rab39b in the other brain regions where we detected synaptic C9orf72, such as the glomerular layer of the olfactory bulb, synapses of the basal ganglia, substantia nigra, and inferior olive, and in the granular layer of the cerebellum (data not shown). For GluR1, we found a significant increase in fluorescence intensity in the entire dorsal hippocampus (*p* < 0.05; Fig. 5f), indicating that C9orf72 is required for the proper regulation of hippocampal GluR1 levels in vivo.

## Discussion

The elucidation of C9orf72 function remains a crucial research endeavor. The present study explored the effect of in vivo knockout of C9orf72 on pre- and post-synaptic protein expression of C9orf72 interactors such as Smcr8 and Rab family proteins, and on post-synaptic receptor levels. Here we show both biochemical and immunohistochemical evidence that C9orf72 localizes to both the pre- and post-synaptic compartment in the mouse forebrain. Our biochemical results also showed that Smcr8 is present in both the pre- and post-synaptic compartments, and recapitulated previous findings showing that loss of C9orf72 expression results in complete ablation of Smcr8 protein expression in the forebrain (Ugolino, 2016). Our immunoblot studies further showed that Rab3a, Rab5, and Rab11 were localized to the pre-synaptic compartment, and that Rab39b is present in both the pre- and post-synapse. In C9-KO mice, PSDs in the hippocampus demonstrated a concomitant decrease in Rab39b levels with increased GluR1 when compared with C9-WT mice. However, we could not detect any protein level changes between C9-WT and C9-KO mice for GluR2 and NMDAR1 in PSDs. Overall, these results suggest that post-synaptic localization of C9orf72 together with its Rab GTPase Rab39b are important for the regulation of glutamatergic receptor levels in vivo.

Interestingly, loss-of-function *RAB39B* mutations are causative of both X-linked mental retardation, showing comorbidity with autism spectrum disorder and epilepsy [19, 58], and early onset Parkinson’s disease with synucleinopathy [58]. RAB39B is important for the coordination of AMPA receptor subunit (GluR1–4) composition and trafficking to the post-synaptic membrane [19, 36]. Here, we demonstrated the co-detection of Rab39b with C9orf72 in the hippocampal mossy fiber region of the mouse forebrain, which is similar to a previous study showing RAB39B overlap with C9orf72 in human iPSC-derived motor neurons [16]. It has also been shown that knockdown of Rab39b in primary neuron cultures results in increased GluR1 trafficking to dendrites [36]. The current study found that a decrease in Rab39b coincides with increased GluR1 in hippocampal PSDs of C9-KO mice. Hence, this work provides in vivo evidence for a link between post-synaptic C9orf72 and its effect on the protein levels of Rab39b and GluR1 in PSDs.

Although C9-KO mice do not demonstrate an overt behavioural phenotype, we showed that loss of the C9orf72 gene leads to alterations in AMPA subunit levels in the murine hippocampus. AMPA receptor/glutamatergic-associated excitotoxicity is a widely studied disease mechanism underlying ALS [5], FTD [7], and ALS/FTD [49]. In addition to increased GluR1 levels demonstrated in C9-KO mice here, upregulation of GluR1 has been observed in other models, such as cultured neurons from SOD1^G93A^ [46] and TDP43^A315T^ [25] transgenic mice that also showed heightened sensitivity to glutamate. Furthermore, increased sensitivity to glutamate has been observed in iPSC-derived neurons from C9-ALS/FTD patients [15, 45, 47]. Our current findings are in line with a study demonstrating an increase of GluR1 transcript and protein levels with a decreased GluR2 level in iPSC-derived motor neurons from C9-ALS patients [45]. While we did not observe an alteration in GluR2 levels in PSD preparations from C9-KO mice, our results indicate that loss of the C9orf72 gene has specific brain region and synaptic subtype effects on AMPA receptor subunit composition in vivo. Another study showed increased GluR1 and NMDAR1 in human C9-ALS post-mortem motor cortices by immunofluorescence and in PSD preparations [47]. In partial agreement with our study, this study also showed that GluR1 was increased in spinal cord motor neurons from C9-KO mice [47]. More recently NMDAR1 and GluR6/7 levels were shown to be increased in the hippocampus of C9orf72 heterozygous mice [48], indicating that decreasing C9orf72 expression is sufficient to alter glutamatergic receptor levels in vivo. Collectively, the results of these studies and our own converge on glutamatergic excitotoxicity as a potentially important aspect of disease mechanism in both the brain and spinal cord of C9-ALS/FTD.

## Conclusion

The present study revealed C9orf72 expression to be an important factor in post-synaptic receptor expression in vivo. We demonstrate a novel localization of C9orf72 to PSDs together with its interactor Smcr8. We also show that loss of C9orf72 in mice results in decreased Rab39b expression and increased GluR1 levels in the hippocampus. Future studies will assess the functional association between post-synaptic C9orf72 and Rab39b protein levels on AMPA receptor trafficking. A better understanding of glutamatergic excitotoxicity in C9-ALS/FTD and in non-C9 ALS/FTD may lead to novel therapeutic development.

## Additional file

**Additional file 1: Table S1.** Primary antibodies used in the current study. **Figure S1.** Rab39b and GluR1 protein levels are unchanged in S1 fraction between C9-WT and C9-KO mice. Bar plots of mean S1 immunoblot band densities for (GAPDH); Ras-related protein Rab39b; Glutamate receptor 1 (GluR1). y-axis = relative density; error bars = standard deviation. All pairwise comparisons are not significant.

## Data Availability

Not applicable.
