# Supplementary material for: Synaptic localization of C9orf72 regulates post-synaptic glutamate receptor 1 levels
Source: Acta Neuropathol Commun. 2019 Oct 24;7:161. doi: 10.1186/s40478-019-0812-5 (PMC6813971; doi:10.1186/s40478-019-0812-5)
Supplement: Supplementary file 1 — Additional file 1: Table S1. Primary antibodies used in the current study. Figure S1. Rab39b and GluR1 protein levels are unchanged in S1 fraction between C9-WT and C9-KO mice. Bar plots of mean S1 immunoblot band densities for (GAPDH); Ras-related protein Rab39b; Glutamate receptor 1 (GluR1). y-axis = relative density; error bars = standard deviation. All pairwise comparisons are not significant. [file 40478_2019_812_MOESM1_ESM.docx]

**Additional file 1**

**Table S1.** Primary antibodies used in the current study.

| **Antibody** | **Manufacturer** | **Catalog #** | **Species/clonality** | **Appn** | **Dilution** |
| --- | --- | --- | --- | --- | --- |
| C9orf72 | GeneTex | GTX634482 | Ms monoclonal | WB, IF | 1000, 5000 |
| GAPDH | Abcam | ab8245 | Ms monoclonal | WB | 1000 |
| Gfap | EMD Millipore | MAB360 | Ms monoclonal | WB | 1000 |
| GluR1 | EMD Millipore | AB1504 | Rb polyclonal | WB, IF | 1000, 200 |
| GluR2 | EMD Millipore | MAB397 | Ms monoclonal | WB | 1000 |
| Nefl | Sigma Aldrich | N5139 | Ms monoclonal | WB | 1000 |
| NMDAR1 | Novus Biologicals | NB300-118 | Ms monoclonal | WB | 1000 |
| PSD95 | Abcam | ab18258 | Rb polyclonal | WB | 1000 |
| PSD95 | Abcam | ab2723 | Ms monoclonal | IP | 200 |
| PSD95 | ThermoFisher | 51-6900 | Rb polyclonal | IF | 200 |
| Rab3a | Synaptic Systems | 107003 | Rb polyclonal | WB | 1000 |
| Rab5 | Santa Cruz | sc-46692 | Ms monoclonal | WB | 1000 |
| Rab11 | BD Transduction | 610657 | Ms monoclonal | WB | 1000 |
| Rab39b | Proteintech | 12162-1-AP | Rb polyclonal | WB, IF | 1000, 2000 |
| Smcr8 | Abcam | ab202283 | Rb polyclonal | WB | 1000 |
| Synaptophysin (Syp) | Synaptic Systems | 101011 | Ms monoclonal | WB | 1000 |
| Synaptoporin (Synpr) | Synaptic Systems | 102002 | Rb polyclonal | IF | 2000 |
| TDP43 | Proteintech | 10782-2-AP | Rb polyclonal | WB | 1000 |

Ms = mouse; Rb = rabbit; Appn = application

**
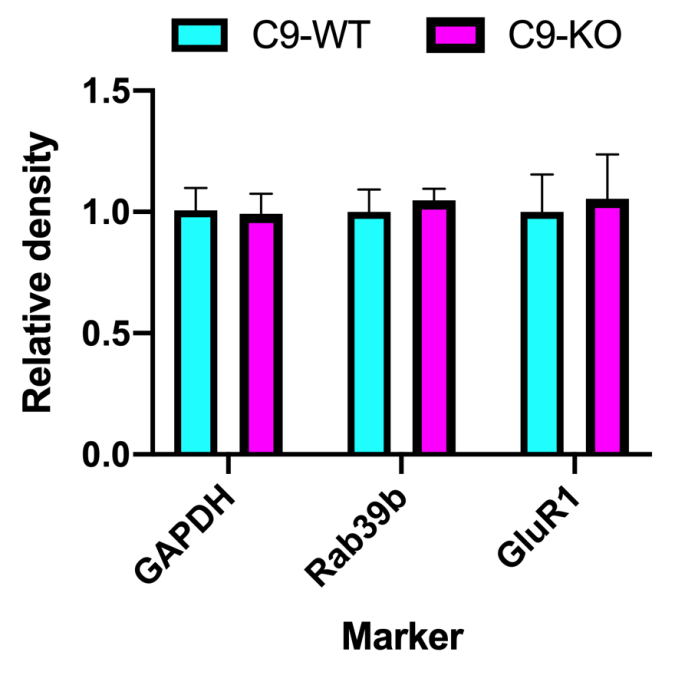
**

**Figure S1. Rab39b and GluR1 protein levels are unchanged in S1 fraction between C9-WT and C9-KO mice.** Bar plots of mean S1 immunoblot band densities for (GAPDH); Ras-related protein Rab39b; Glutamate receptor 1 (GluR1). y-axis = relative density; error bars = standard deviation. All pairwise comparisons are not significant.
